# Ethics and accountability for clinical trials

**DOI:** 10.1038/s41393-024-00980-z

**Published:** 2024-03-18

**Authors:** Tanya A. Barretto, Wolfram Tetzlaff, Judy Illes

**Affiliations:** 1 Neuroethics Canada, Division of Neurology, Department of Medicine, University of British Columbia, Vancouver, BC Canada; 2 International Collaboration on Repair Discoveries (ICORD), University of British Columbia, Vancouver, BC Canada

**Keywords:** Clinical trials, Translational research

## Abstract

In May 2023, a disclaimer posted on ClinicalTrials.gov dismisses accountability for the accuracy of registered information. For spinal cord injury, inconsistencies in intervention classification, phase designation, and lack of study protocols and results threaten the integrity of the database and put users at risk. An investment in what the resource should be rather than what it is not will give it the authority commensurate with the requirements for its regulatory use and informed decision-making for prospective trial participants.

## Main text

Clinical trial registration on ClinicalTrials.gov is required for trials under the jurisdiction of the Food and Drug Administration (FDA), those funded by the NIH, and trial results published in journals under the umbrella of the International Committee of Medical Journal Editors (ICMJE). If registration is mandatory, then curation to ensure accurate reporting is necessary. On May 25, 2023, the United States National Library of Medicine at the National Institutes of Health (NIH) posted a disclaimer on ClinicalTrials.gov effectively disowning accountability for errors or omissions of data uploaded to its site. It states:

*“The U.S. government does not review or approve the safety or science of all studies listed on this website. The study sponsor or investigator submits information about their study to ClinicalTrials.gov and is responsible for the safety, science, and accuracy of any study they list.”* (ClinicalTrials.gov)

The stated purpose of the ClinicalTrials.gov database, *“… is to provide information about clinical research studies to the public, researchers, and health care professionals*”, combined with the new disclaimer is paradoxical to its efforts to “*support laws, regulations, and policies that require sponsors and investigators to publicly share information about clinical trials, including results*”. Rather than addressing areas for improvement through monitoring or a standardized approval process, the May update trivializes the information ClinicalTrials.gov professes to offer, undermines regulatory and publication requirements, and is offensive to all who rely on it.

Significant gaps in the ClinicalTrials.gov database have been documented in the past [1]. We are particularly concerned about clinical trials for people living with spinal cord injury (SCI) for whom biomedical innovation is regularly featured in news media, and who may turn to the database for scientific details and opportunities for enrollment in trials themselves. Each year, the life-altering condition of SCI affects between 250,000 and 500,000 people worldwide [2]; with life-time healthcare costs ranging from US$1.3–5.8 million in North America [3], creating a dire unsolved need for effective, population-wide interventions.

The lack of oversight and enforcement permits inconsistencies and incomplete information to be posted on the database. Medically vulnerable participants are left to decipher variances in definitions, such as the timing of interventions. For example, the recorded timeframe for acute injuries spans a few hours to a few months post-injury (e.g., NCT02260713, NCT04331405, NCT01694927), and for chronic injuries ranges 5 to 18 months and in some cases 20 years (NCT01005615). Some entries do not define the time window of recruitment at all. While a lack of consensus on timing definitions may mirror the research literature where a surgical intervention can define acute as <24 h and a biological intervention as <12 h, we could not determine any such trend from our examination of over 400 studies.

While the labeling of interventions for some trials are correct, such as TCA Cellular Therapy (USA) NCT01162915 transfer of bone marrow derived stem cells for the treatment of SCI, others are inconsistent. Some stem cells trials may be categorized as a drug, genetic, or procedural intervention (Table 1) rather than the biological one that it is. Trials with Botox/Botulin toxin A are categorized as both as a biological intervention and as a drug intervention. Stem cells are unequivocally a biological product and should be categorized as such.

**Table 1:** Examples of stem cell and botulin toxin A trials for SCI with inconsistent intervention categorization.

ID	Title	Intervention/Treatment		ClinicalTrials.gov category	Discrepancy
*Stem Cells*					
NCT01772810 (USA)	Safety study of human spinal cord-derived neural stem cell transplantation for the treatment of chronic SCI	Human spinal cord stem cells		Drug	Categorized as a drug intervention
NCT03505034 (China)	Intrathecal transplantation of UC-MSC in patients with late stage of chronic spinal cord injury	Umbilical cord mesenchymal stem cells		Drug	Categorized as a drug intervention
NCT05054803 (Spain)	Cell therapy for chronic traumatic cervical incomplete spinal cord injury	WJ-MSC (XCEL-UMC-BETA) Placebo		Drug Drug	Categorized as a drug intervention
NCT01769872 (Korea)	Safety and effect of adipose tissue derived mesenchymal stem cell implantation in patients with spinal cord injury	Autologous adipose tissue derived MSCs (mesenchymal stromal cell) transplantation		Procedure	Categorized as a procedural intervention
NCT05671796 (Brazil)	Autologous bone marrow stem cell transplantation in patients with subacute spinal cord injury	Mesenchymal stem cell transplantation Placebo		Genetic Other	Categorized as a genetic intervention Ambiguous categorization
*Botulin Toxin A*					
NCT02660138 (USA)	Dysport® Treatment of urinary incontinence in adults subjects with neurogenic detrusor overactivity (NDO) due to spinal cord injury or multiple sclerosis – Study 1		Botulinum toxin type A Placebo	Biological Drug	Categorized as a biological intervention Categorized as a drug intervention
NCT01911377 (Canada)	Botulinum toxin type A for treating allodynic pain in SCI and MS	Botulinum Toxin Type A Normal saline for injection		Drug Drug	Categorized as a drug intervention Categorized as a drug intervention
NCT02052024 (USA)	Myobloc Atrophy study	Botox MYOBLOC		Drug Drug	Categorized as drug interventions

Examples are chosen to illustrate the range of countries with registered trials. Entries are verbatim.

The Not Applicable Phase category, defined as “*trials without FDA-defined phases, including trials of devices or behavioral interventions*” presents further ambiguity. It accounts for a majority of SCI clinical trial studies [1]. However, there is a lack of clarity about what type of intervention qualifies for a Not Applicable Phase study versus Phase I, or if a successful trial in the Not Applicable Phase moves to a Phase I or to directly a Phase IV designation. Two biomaterial scaffolds that are in trials illustrate this concern: one is registered in the Not Applicable Phase and categorized as a device; the other is registered in Phase II and categorized as a biological intervention (Table 2). This opacity in the categorization of intervention or phase is a serious barrier to scientific reliability and accountability.

**Table 2:** Comparison of two biomaterial scaffold trials with varying intervention and Phase categorizations.

ID	Title	Intervention/ Treatment	ClinicalTrials.gov category	Phase
NCT02138110	The INSPIRE study: Probable benefit of the neuro-spinal scaffold for treatment of AIS A thoracic acute spinal cord injury	Neuro-Spinal Scaffold	Device	Not Applicable
NCT02688049	NeuroRegen™ Scaffold combined with stem cells for chronic spinal cord injury repair	NeuroRegen scaffold/mesenchymal stem cells transplantation NeuroRegen scaffold/neural stem cells transplantation	Biological	Phase I Phase II
NCT02688062	NeuroRegen™ Scaffold with bone marrow mononuclear cells transplantation vs intradural decompression and adhesiolysis in SCI	NeuroRegen scaffold with BMMCs transplantation Surgical intradural decompression and adhesiolysis	Biological Procedure	Phase I Phase II

Entries are verbatim.

The FDA calls for mandatory reporting of results and study protocols to ClinicalTrials.gov for applicable clinical trials [4], yet the majority of completed trials do not have results posted on the database [1] and the recent disclaimer further nullifies this requirement. The absence of published results pertaining to reported adverse events is a critical barrier to informed decision-making. Uncurated and vague criteria such as leaving undefined exclusion criteria to investigator discretion pose ethical challenges to transparency and inclusivity.

SCI trials are suspended, terminated, or withdrawn due to low enrollment numbers [5, 6]. People living with SCI rely heavily on online sources for information and the trustworthiness of available information is second only to interest [7]. Both are compromised when information is poor or inaccurate, creating a void that can be filled by unscrupulous sources leading to misinformation and mistrust in science. This is a violation of any code of ethics governing research, and ultimately will translate to hesitation to take up interventions that are brought to market. A SCI specific clinical trial database (https://scitrialsfinder.net/trials) has been created to meet the needs of the community. If every patient group had to resort to hosting its own trials tracking site, the proliferation of inefficiencies, inaccuracies, and dubiety would be extraordinary.

Vague wording in the 2013 amendments to the Declaration of Helsinki creates ambiguity around the responsible party for ensuring post-trial access in low- and middle-income countries demonstrating a further waning from ethical principles meant to protect vulnerable participants [8]. The FDA does not require compliance with the Declaration of Helsinki for protocols under their jurisdiction but implemented overseas. Although post-trial access is normally identified in the consent protocols, listing them on the ClinicalTrials.gov database would significantly enhance transparency.

Oversight and enforcement of registered studies is essential for the reliability and utility of the ClinicalTrials.gov database, and accountability for the accuracy and completeness of study information is vital to fulfilling its purpose. Clear definitions and consistency across the type of interventions and categorization of trial phase will provide clarity and reliability of available information; monitored transparency about adverse events will bring new research to improve the quality-of-life of people living with SCI, clinical outcomes, and addressing the related economic burden of SCI (Table 3).

**Table 3:** Summary of observations and recommendations.

1. **Consistency across intervention type**: The primary challenge in understanding the landscape of available trials is the ambiguous classification of intervention type. Clear and consistent definitions must be implemented and curated.
2. **Transparency around phase designations**: The majority of SCI studies are registered in the Not Applicable Phase that is not in line with the defined Phases. The reliability and trustworthiness of the scientific status of these trials is undermined until the gaps for this category are addressed.
3. **Transparency around adverse events**: The currently disclaimed accountability applies to the reporting of adverse events alongside other currently permitted lapses in trial registration and updating. Informed decision-making for trial participation is impossible in this context.

Although we acknowledge that reviewing the safety and science of all studies is a high bar to reach, oversight for consistency and standardization in terminology and categorization of an intervention is a must for the database to fulfill its stated purpose. Rather than hiding behind a disclaimer, an investment in Clinicaltrials.gov that is commensurate with the gravitas of the requirements for engaging with it will empower contributors, revive the usefulness of this important resource, and support the autonomy and decision-making of trial participants.
